# Outcomes of low-dose immune tolerance induction with single-dose rituximab in severe hemophilia A: a single-center retrospective experience

**DOI:** 10.3389/fimmu.2026.1829007

**Published:** 2026-04-30

**Authors:** Shaoyu Yin, Hongli Mu, Wen Yang, Zeping Zhou

**Affiliations:** Department of Hematology, the Second Affiliated Hospital of Kunming Medical University, Kunming, China

**Keywords:** immune tolerance induction, low-dose, rituximab, severe hemophilia A, single-dose

## Abstract

**Background:**

Low-dose immune tolerance induction (ITI) combined with standard-dose rituximab-based immunosuppression (LD-ITI + RTX-IS) is an effective strategy for factor VIII (FVIII) inhibitor eradication in severe hemophilia A with poor-risk prognostic factors for ITI, but optimal rituximab dosing remains undefined. Prolonged rituximab exposure increases adverse effects and financial burden.

**Aim:**

To describe the outcomes and tolerability of LD-ITI combined with single-dose rituximab (LD-ITI + SD-RTX-IS) for FVIII inhibitor eradication in severe hemophilia A.

**Methods:**

A single-center retrospective cohort study was conducted in patients with severe hemophilia A and inhibitors who received LD-ITI + SD-RTX-IS. Treatment comprised FVIII (50 IU/kg every other day), rituximab (375 mg/m², single dose), and prednisone (1 mg/kg daily, tapering). Success was defined as inhibitor titer <0.6 BU/mL and FVIII recovery ≥66%.

**Results:**

A total of 16 patients (median age 17 years) were enrolled, comprising 8 adults and 8 children. Eleven of 16 patients (68.8%) achieved successful inhibitor eradication with a median time of 9.3 months (IQR: 2.8–15.2; range: 1.8–18.6); median FVIII recovery was 82%. The monthly bleeding rate decreased by 84.2% from pre-ITI (median: 4 episodes) to during ITI (median: 0.66 episodes), with a corresponding reduction in monthly joint bleeds from 3 to 0.46 episodes. At 24-month follow-up, no recurrence was documented. Adverse events were predominantly mild (rituximab-related infusion reactions, n=2; fever/pneumonia, n=4).

**Conclusions:**

In this cohort, LD-ITI + SD-RTX-IS was associated with an inhibitor eradication rate consistent with prior LD-ITI + RTX-IS reports, using less rituximab. These preliminary findings warrant further prospective evaluation.

## 1 Introduction

Antibodies (inhibitors) against coagulation factor VIII (FVIII) develop in 25%–30% of patients with severe hemophilia A. Currently, immune tolerance induction (ITI) is the only proven strategy for eliminating inhibitors (1). Current guidelines recommend risk-stratified ITI dosing, incorporating factors such as the pre-ITI inhibitor titer, historical peak inhibitor titer, inhibitor titer during ITI, and the time interval after ITI initiation (2–5). In good-risk patients [pre-ITI titer <40 Bethesda Units (BU)/mL, historical peak titer <100 BU/mL, or titer during ITI <40 BU/mL], both low-dose (LD; 50 IU/kg every other day) and high-dose (HD; 200 IU/kg/day) regimens achieve comparable success rates (6, 7). Poor-risk patients (pre-ITI titer ≥40 BU/mL, historical peak titer ≥100 BU/mL, or titer during ITI ≥40 BU/mL) should receive intermediatedose (MD; 100 IU/kg/day) or high-dose ITI to optimize outcomes (6, 7).

In China, MD- or HD-ITI, which is recommended for poor-risk patients, is financially unaffordable for most families. To address this challenge, Wu et al. demonstrated that low-dose ITI combined with standard-dose rituximab-based immunosuppression (LD-ITI + RTX-IS) is an effective strategy for inhibitor eradication in patients with poor-risk prognostic factors for ITI (2, 8–11). The regimen is as follows: FVIII concentrate at a dosage of 50 IU/kg every other day, combined with rituximab at a dose of 375 mg/m² intravenously once per week (maximum 600 mg per dose) for a total of 4 doses, along with prednisone at 2 mg/kg daily for 1 month (maximum 60 mg per day, tapering over 3 months) (8–11).

However, the optimal rituximab dose for LD-ITI combination therapy remains undefined. This knowledge gap is concerning because prolonged rituximab exposure increases the risk of adverse effects, such as infusion reactions and immunosuppression-related complications. Furthermore, the financial burden of this regimen is substantial. For a 50 kg patient, each ITI course in this regimen costs $4, 500 for rituximab. Additionally, the regimen requires intravenous immunoglobulin (IVIG; 200 mg/kg per month for 6 months) to prevent infection during the 6 months following rituximab treatment, which further increases the cost of one ITI course by $1228 (12). In resource-limited settings like China, this is undoubtedly a significant expense. We therefore hypothesized that reducing rituximab to a single dose would maintain comparable efficacy while improving safety and lowering costs, and conducted a retrospective cohort study evaluating LD-ITI with a single-dose RTX-IS (LD-ITI + SD-RTX-IS) for FVIII inhibitor eradication in patients with severe hemophilia A and poor-risk ITI prognostic factors.

## 2 Methods

This single-center retrospective cohort study was conducted at the Second Affiliated Hospital of Kunming Medical University. Written informed consent was obtained from the participants and parent guardians in case of minors. It was approved by the Ethics Committee of the Second Affiliated Hospital of Kunming Medical University (approval no. 2025-282).

### 2.1 Patients

We included patients who underwent LD-ITI + SD-RTX-IS for inhibitor eradication from July 2022 to August 2023. The last follow-up was completed by March 1, 2026. We collected patients’ characteristics, including their *F8* variant types, inhibitor titers, various interval-times, bleeding episodes, and ITI outcomes.

### 2.2 Inclusion and exclusion criteria

Inclusion criteria: (i) any age; severe HA (FVIII <1 IU/dL prior to inhibitor development); (ii) first ITI attempt; (iii) having received the LD-ITI + SD-RTX-IS regimen for inhibitor eradication.

Exclusion criteria: (i) congenital or acquired bleeding disorder(s) other than HA; (ii) presence of concomitant immunological disease(s).

### 2.3 Treatment strategy

Treatment comprised inhibitor clearance and hemostatic management.

To reduce the cost of immunosuppressants and minimize their immunosuppressive effects, the frequency of rituximab administration was reduced, and the dosage of prednisone was adjusted. Specifically, the regimen includes recombinant [r]FVIII (omfiloctocog alfa, SCT800) concentrate at a dosage of 50 IU/kg every other day, combined with rituximab at 375 mg/m² intravenously (maximum of 600 mg) for a single dose, and prednisone at 1 mg/kg daily for 1 month (maximum of 60 mg per day, tapering over 3 months).

The principles of adding SD-RTX-IS in patients with poor-risk prognostic factors were as follows: (i) patients with a historical peak inhibitor titer of ≥100 BU/mL or a titer of ≥40 BU/mL at the onset of ITI should be given SD-RTX-IS; (ii) patients on LD-ITI alone should be switched to LD-ITI + SD-RTX-IS if their peak titer reaches ≥40 BU/mL during ITI or if the inhibitor decline is <20% in the first 3 months following the initial peak titer during ITI (3).

To mitigate corticosteroid-related adverse effects, all patients received gastroprotective agents, calcium supplementation, and potassium supplementation concurrently.

Breakthrough bleeding episodes occurring during ITI when the inhibitor titer was ≥5 BU/mL were managed with domestic prothrombin complex concentrate (50 IU/kg every 8–12 hours) or recombinant activated factor VII (90 μg/kg every 4–6 hours). Once the inhibitor titer declined to <5 BU/mL, bleeding was controlled using rFVIII concentrate at 50 IU/kg.

### 2.4 Inhibitor detection and definition of success

Inhibitor titer measurement was performed at the Second Affiliated Hospital of Kunming Medical University using the Bethesda assay (Nijmegen modification). Inhibitor titer was monitored every 2 weeks until a steady inhibitor titer decline was observed, and then monthly. *In vivo* FVIII recovery was performed once the inhibitor titer was negative twice consecutively at least two weeks apart.

Success: achieving negative inhibitor titer (<0.6 BU/mL) twice consecutively at least two weeks apart and FVIII recovery ≥66% of expected. Success here does not imply “tolerance” given that a FVIII half-life (t_1/2_) >6 h was not included in the definition.

Non-success: failure to achieve the success criteria.

Relapse defined as recurrence of inhibitor to ≥0.6 BU/mL after the patient had achieved success.

### 2.5 Subsequent treatments

Once the patient achieved success, the FVIII dose would be reduced slowly to 25 IU/kg three-times/week for continuing prophylaxis. None of the patients received non-factor therapy as prophylactic treatment.

### 2.6 Statistics

Categorical variables are expressed as frequencies and percentages. Continuous variables are expressed as median values with interquartile range (IQR) and full range (minimum–maximum). All statistical analyses were performed using SPSS 26.0. The cumulative incidence of success was estimated by the method of Kaplan-Meier method.

## 3 Results

### 3.1 Patient characteristics

**Figure 1:**
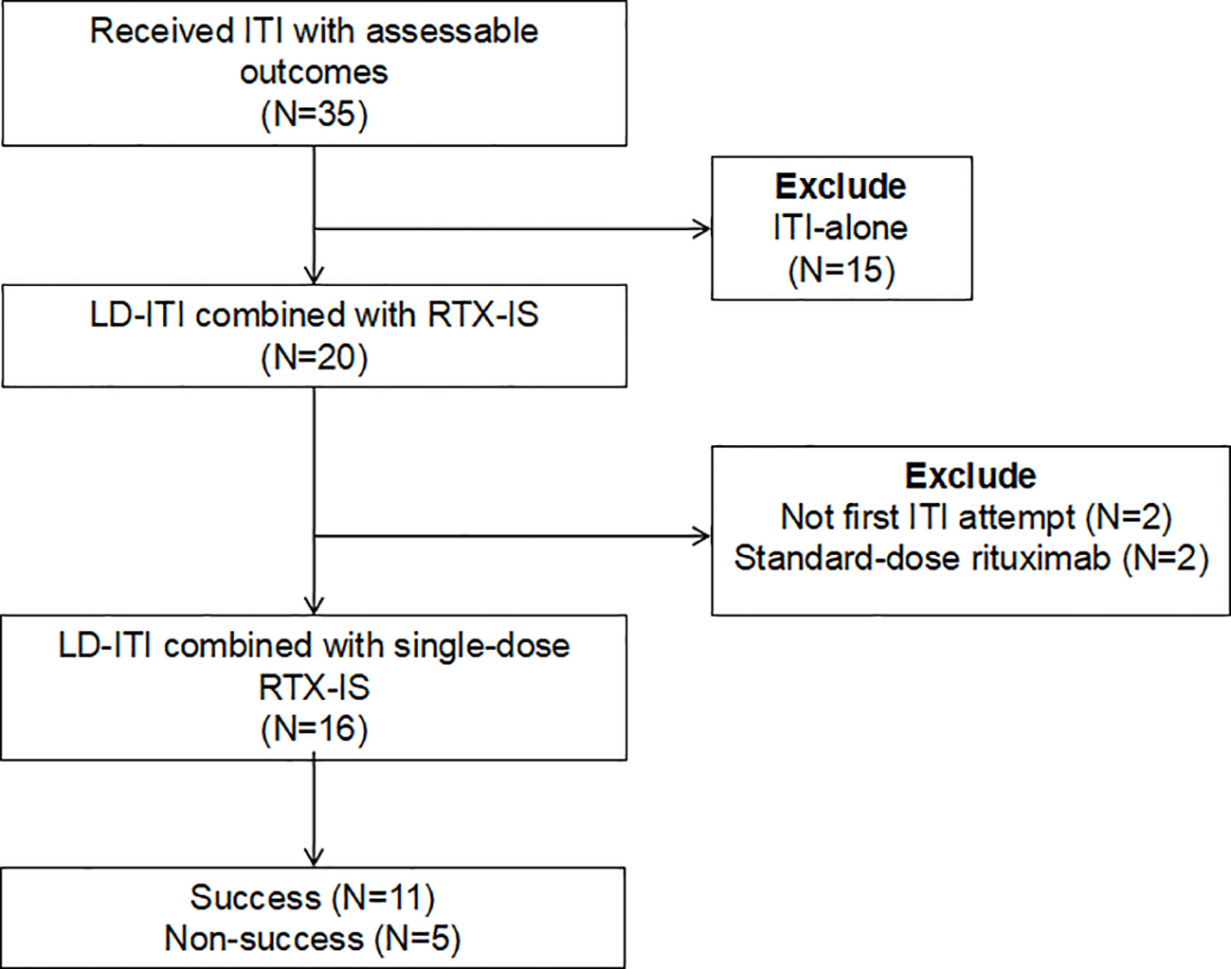
Patient selection flowchart. RTX-IS was indicated in patients with a historical peak inhibitor titer ≥100 BU/mL or a titer ≥40 BU/mL at ITI onset. Patients initially on LD-ITI alone were switched to LD-ITI + RTX-IS if their peak titer reached ≥40 BU/mL during ITI or if inhibitor decline was <20% within the first 3 months following the initial peak titer. ITI, immune tolerance induction; LD-ITI, low-dose ITI; RTX-IS, rituximab-based immunosuppression; BU, Bethesda Units.

Patient selection flowchart is shown in **Figure 1**. This study included a total of 16 patients who received LD-ITI + SD-RTX-IS. The median age at the start of ITI was 17 years (IQR: 13.5–36.5; range: 4–57), with 8 adults and 8 children (**Table 1**). All patients received on-demand therapy before ITI initiation. The median duration from the onset of ITI to the last follow-up was 32.2 months (IQR: 31.2–36.1; range: 27.5–44.1). All 16 patients had pathogenic *F8* variants: 1 frameshift, 8 large deletions, 5 nonsense variants, and 2 intron 22 inversions (**Supplementary Table 1**). The median historical peak titer before ITI was 113.9 BU/mL (IQR: 38.3–222.9; range: 10.4–409.6). The median titer at the start of ITI was 24.2 BU/mL (IQR: 6.2–66.9; range: 1.0–409.6), of which 75% (12/16) were high titer (≥5 BU/mL), and the median peak titer during ITI was 33.5 BU/mL (IQR: 10.4–226.6; range: 1.1–4505.6). The median interval from the diagnosis of inhibitors to the initiation of ITI was 12.3 months (IQR: 1.3–33.6; range: 0–112.6). Ten patients received SD-RTX-IS concurrently with ITI initiation. Six patients were switched from LD-ITI alone to LD-ITI + SD-RTX-IS at a median of 5.1 months (IQR: 4.0–7.2; range: 3.2–11.3) after ITI initiation due to inadequate response.

**Table 1:** Patient baseline demographics and clinical characteristics.

Patients (N = 16)	
Age group, n (%)	
<18 years	8 (50)
≥18 years	8 (50)
Number of patients tested for *F8* variant, n (%)	
Nonsense	5 (31.2)
Large deletion	8 (50.0)
Intron 22 inversion	2 (12.5)
Frameshift	1 (6.3)
Age at start of ITI (years), median (IQR, range)	17 (13.5–36.5, 4–57)
Time interval (months), median (IQR, range)	
From inhibitor diagnosis to ITI start	12.3 (1.3–33.6, 0–112.6)
From ITI start to IS use	5.1 (4.0–7.2, 3.2–11.3)^a^
Inhibitor titer (BU/mL), median (IQR, range)	
Peak historical inhibitor titer	113.9 (38.3–222.9, 10.4–409.6)
Pre‐ITI inhibitor titer	24.2 (6.2–66.9, 1.0–409.6)
Peak inhibitor titer during ITI	33.5 (10.4–226.6, 1.1–4505.6)
Monthly bleeds/monthly joint bleeds, median (IQR, range)	
Pre-ITI	4 (3–5, 2–10)/3 (3–4, 1–8)
During-ITI	0.66 (0.49–0.93, 0.36–1.43)/0.46 (0.38–0.74, 0.27–1.43)
Annual bleeding rate/annual joint bleeding rate, median (IQR, range)	
Pre-ITI	48 (36–60, 24–120)/36 (36–48, 12–96)
During-ITI	7.56 (5.9–11.1, 4.32–17.14)/5.50 (4.4–9.3, 3.28–17.14)

ITI, immune tolerance induction; IQR, interquartile range; IS, immunosuppression.

a. Six patients were switched from ITI alone to ITI combined with immunosuppression: four due to a peak inhibitor titer of ≥40 BU/mL during ITI, and two due to inadequate inhibitor titer reduction (<20% within 3 months).

### 3.2 Treatment measures

#### 3.2.1 Clearing inhibitors

**Figure 2:**
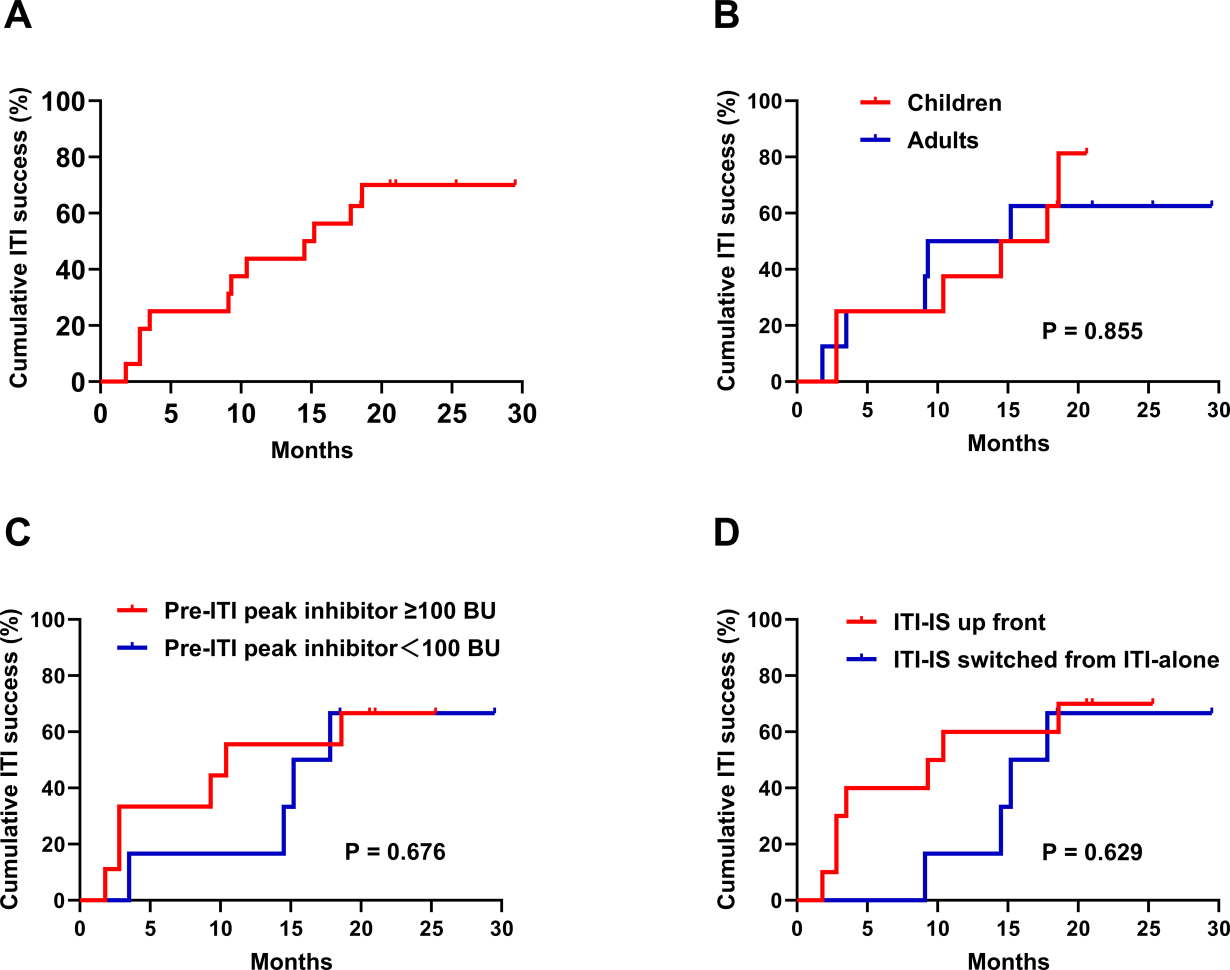
Kaplan-Meier plots summarizing the observed time to inhibitor eradication (inhibitor titer <0.6 BU/mL and factor VIII recovery ≥66% of expected) of ITI. **(A)** Kaplan-Meier plot shows the time-to-success. **(B)** Kaplan-Meier plot shows the time-to-success between adults and children. **(C)** Kaplan-Meier plot shows the time-to-success between pre-ITI peak inhibitor titer ≥100 BU/mL and <100 BU/mL. **(D)** Kaplan-Meier plot shows the time-to-success between the ITI-IS upfront group and the ITI-IS switched from ITI-alone group. Because rituximab was administered concurrently at ITI initiation in ten patients and added following inadequate response in six others, patients followed heterogeneous treatment paths; these curves reflect observed patterns in this cohort and should not be interpreted as representing the effect of a single fixed intervention or as supporting direct comparison with other ITI protocols. ITI, immune tolerance induction; IS, immunosuppression.

In our cohort, 11 patients (11/16, 68.8%) achieved success within a median of 9.3 months (IQR: 2.8–15.2; range: 1.8–18.6) (**Table 2**, **Figure 2**). The median FVIII recovery in these 11 patients was 82.0% (IQR: 73.0%–97.0%; range: 70.5%–157.0%). Among these, six patients underwent half-life (t_1/2_) testing, all of whom demonstrated a t_1/2_ of >6 hours [Median: 8 hours (IQR: 7.9–8.9; range: 7.2–10.1)]. Among the five patients who did not achieve success, two maintained low inhibitor titer (<5 BU/mL), allowing them to receive on-demand FVIII replacement therapy in subsequent treatments.

**Table 2:** Immune tolerance induction outcomes.

Therapeutic efficacy	Patients (N = 16)	ITI-IS upfront subgroup (n=10)	ITI-alone switched to ITI-IS subgroup (n=6)
Success, n (%)	11 (68.8)	7 (70.0)	4 (66.7)
Non-success, n (%)	5 (31.2)^a^	3 (30.0)	2 (33.3)
Time to success (months), median (IQR, range)	9.3 (2.8–15.2, 1.8–18.6)^b^	3.5 (2.8–10.4, 1.8–18.6)	14.8 (10.4–17.1, 9.1–17.8)
Time from rituximab addition to ITI success (months), median (IQR, range)	7.9 (4.4–12.6, 3.9–13.5)	-	7.9 (4.4–12.6, 3.9–13.5)
FVIII recovery (%), median (range)	82.0 (73.0–97.0, 70.5–157.0)	82 (76.0–102.0, 70.5–157.0)	83.5 (71.5–96.2, 71.0–97.0)
FVIII half-life ≥6 hours, n (%)	6 (37.5)	3 (30.0)	3 (50.0)
FVIII half-life (hours), median (IQR, range)	8 (7.9–8.9, 7.2–10.1)	8 (7.2–8.9, 7.2–8.9)	8 (7.9–10.1, 7.9–10.1)
Follow-up period after ITI success (months), median (IQR, range)	24.0 (20.8–28.4, 11.3–29.6)	24.0 (21.8–28.4, 11.3–29.6)	23.8 (19.1–28.4, 18.5–28.9)
Relapse, n (%)	0 (0)	0 (0)	0 (0)

ITI-IS, immune tolerance induction combined with immunosuppression; FVIII, factor VIII; IQR, interquartile range; −, not available.

a. Two patients had an inhibitor titer < 5 BU/mL.

b. For the 11 successful patients and the 2 patients with inhibitor titers < 5 BU/mL, the median treatment duration was 10.4 (range:1.8–29.5) months.

The following subgroup analyses are exploratory and descriptive in nature, intended to generate hypotheses rather than draw inferential conclusions, given the limited sample size of this cohort. Subgroup analyses were performed stratified by patient age (adults vs. children), pre-ITI peak inhibitor titer (≥100 BU/mL vs. <100 BU/mL), and timing of immunosuppressive therapy (ITI-IS upfront vs. ITI-IS switched from ITI-alone subgroup) (**Figure 2**). In the ITI-IS upfront subgroup, 7 of 10 patients (70.0%) achieved ITI success. Six patients were switched from ITI alone to ITI plus immunosuppressive therapy due to inadequate response: four had peak inhibitor titers ≥40 BU/mL and two showed insufficient titer reduction (<20% within 3 months); of these, four achieved ITI success at a median of 7.9 months (IQR: 4.4–12.6; range: 3.9–13.5) after escalation. Despite a longer median time to success in the ITI-IS switched from ITI-alone subgroup than in the ITI-IS upfront subgroup (14.8 vs. 3.5 months), no clear difference in success rate or time to success was observed between the two subgroups in this cohort (P = 0.629). ITI success rates were similar between adult and pediatric patients (62.5% [5/8] vs. 75.0% [6/8]), with median time to success of 9.1 and 12.4 months, respectively; no clear difference was observed between the adult and pediatric subgroups (P = 0.855). Likewise, no clear differences were observed between patients with pre-ITI peak inhibitor titers ≥100 and <100 BU/mL in success rate (66.7% [6/9] vs. 71.4% [5/7]) or time to success (9.1 vs. 14.5 months) (P = 0.867).

#### 3.2.2 Bleeding episodes

A total of 153 breakthrough bleeding episodes occurred during ITI, none of which required hospitalization or resulted in serious hemorrhagic complications. Of the 153 breakthrough bleeding episodes, 112 (73.2%) were joint bleeds, predominantly involving the knee, ankle, and elbow joints, while the remaining 41 episodes (26.8%) consisted of mucocutaneous and muscle bleeds. Each bleeding episode was treated with prothrombin complex concentrate. The monthly bleeding rate significantly decreased from a median of 4 (IQR: 3–5; range: 2–10) episodes pre-ITI to a median of 0.66 (IQR: 0.49–0.93; range: 0.36–1.43) episodes during ITI, representing an 84.2% reduction. The median monthly joint bleeding frequency decreased from 3 (IQR: 3–4; range: 1–8) to 0.46 (IQR: 0.38–0.74; range: 0.27–1.43) bleeds per month during ITI, representing a reduction of 84.7%. Annual bleeding rates and annual joint bleeding rates are presented in **Table 1**.

### 3.3 Relapse

During the median follow-up period of 24.0 months (IQR: 20.8–28.4; range: 11.3–29.6), no recurrence was observed in patients who achieved ITI success.

### 3.4 Adverse events

Adverse events were generally mild and manageable. One patient experienced nausea during rituximab infusion, and another developed a rash, both attributed to rituximab-related infusion reactions and successfully managed with antihistamine therapy. One patient developed fever with chest imaging (CT scan) revealing pneumonia, which was treated with antibiotics and resolved. Three additional patients experienced fever that was managed with antipyretic therapy without further complications. None of the patients developed corticosteroid-related adverse effects.

## 4 Discussion and conclusions

To our knowledge, this is the first study to evaluate LD-ITI + SD-RTX-IS for severe hemophilia A patients with poor-risk inhibitor eradication prognostic factors. The LD-ITI + SD-RTX-IS regimen achieved inhibitor eradication in 68.8% (11/16) of patients, with a median time to successful eradication of 9.3 months (IQR: 2.8–15.2; range: 1.8–18.6). During a median follow-up period of 24.0 months (IQR: 20.8–28.4; range: 11.3–29.6), no patient experienced inhibitor recurrence. Adverse events were predominantly mild and manageable with appropriate supportive care.

Incorporating IS in poor-risk patients helps overcome pre-existing immunological memory (2). FVIII exposure alone during ITI aims to induce T-cell tolerance but is frequently insufficient against established B-cell clones (13). Rituximab addresses this by targeting CD20+ B-cells and depleting plasma cell precursors, which allows ITI to successfully induce tolerance (2, 13).

The LD-ITI + RTX-IS regimen is currently the most widely used treatment strategy in China for hemophilia A patients with poor-risk prognostic factors for inhibitor eradication, with reported success rates of 42.1%–66.7% and median times to success of 10.8–13.6 months (**Table 3**) (8–11). In our cohort, LD-ITI + SD-RTX-IS was associated with an inhibitor eradication rate consistent with prior LD-ITI + RTX-IS reports. Additionally, two patients with low inhibitor titers, although not meeting the formal success criteria, achieved clinically meaningful outcomes that enabled FVIII replacement therapy, further supporting the clinical value of this approach. Nonetheless, well-designed, large-scale, concurrent controlled studies are still required to definitively determine whether the reduction in rituximab dosage affects the success rate and/or prolongs the time to treatment success. The rationale for reducing the rituximab dose stems from the observation that the dose and frequency of rituximab in LD-ITI + RTX-IS regimens have been largely adopted from lymphoma treatment protocols. Given that the lymphocyte burden in immune-mediated diseases is substantially lower than that in lymphoproliferative disorders, we hypothesized that a reduced single dose of rituximab might be sufficient to achieve adequate depletion of aberrantly activated B lymphocytes (14, 15). However, further studies are required to validate this hypothesis.

**Table 3:** Immune tolerance induction regimens for patients with poor prognosis risk factors.

Study	Current study	Li et al. (8)	Li et al. (9)	Li et al. (10)	Li et al. (11)	Unuvar et al. (16)	Zulfikar et al. (17)	Kreuz et al. (20)
Regimen	LD-ITI + SD-RTX-IS	LD-ITI ± IS	LD-ITI + RTX-IS	LD-ITI + RTX-IS	LD-ITI + RTX-IS	LD-ITI	LD-ITI	HD-ITI
Number of patients (n)	16	9	19	27	36	19	12	35
Patients achieving success (%)	68.8 (11/16)	66.7 (6/9)	42.1 (8/19)	66.7 (18/27)	44.4 (16/36)	26.3 (5/19)	33.3 (4/12)	68.6 (24/35)
Median time to achieve success (mo)	9.3 (1.8–18.6)	13.2 (6.5–13.6)	13.6	10.8 (3.1–20.9)	13.6 (3.5–29.9)	6 (3–12)	27.5 (8–54)	6.06 (3.10–11.96)
Monthly bleeding rate	0.66 (0.36–1.43)	NR	NR	NR	0.32 (0–1.50)	NR	NR	NR
Relapsed (%)	0	6.3	0	0	6.3	NR	NR	0
Adverse events	Infection (4) and infusion reactions (2)	Infection (5)	Infection (1)	NR	Infusion reactions (10) and infection (1)	NR	NR	Allergic dermatitis (1), and serious adverse events (4)

LD-ITI + SD-RTX-IS: low-dose Immune Tolerance Induction (factor VIII 50 IU/kg every other day) combined with single-dose rituximab-based immunosuppression, consisting of rituximab 375 mg/m² intravenously (maximum 600 mg) and prednisone 1 mg/kg/day orally for 1 month (maximum 60 mg/day), followed by tapering over 3 months; LD-ITI + RTX-IS: low-dose ITI combined with standard rituximab-based immunosuppression, consisting of rituximab 375 mg/m² intravenously (maximum 600 mg) once weekly for 4 consecutive doses, and prednisone 2 mg/kg/day orally for 1 month (maximum 60 mg/day), followed by tapering over 3 months; LD-ITI: low-dose Immune Tolerance Induction; HD-ITI: high-dose (factor VIII 200 IU/kg/day).

NR, not reported.

Values in parentheses in the “Adverse events” column represent the number of affected patients.

In contrast, LD-ITI alone yields substantially lower success rates of only 26.3%–33.3% in this poor-risk population (16, 17). Regarding rituximab monotherapy, a clinical trial by Leissinger et al. demonstrated that only 3 of 16 patients (18.8%) achieved a major response (inhibitor titer <5 BU/mL without anamnestic rise upon FVIII re-exposure), and only 1 (6.3%) achieved a minor response (18). Similarly, in a national cohort of patients receiving salvage rituximab monotherapy, none achieved complete sustained remission, whereas 58.3% of patients receiving ITI combined with rituximab achieved complete sustained remission (19). These findings collectively indicate that neither LD-ITI alone nor rituximab monotherapy is sufficient for inhibitor eradication in poor-risk patients, and that combination therapy can optimize outcomes.

The observed success rate of 68.8% in our cohort is numerically similar to that reported for HD-ITI (68.6%), although the median time to success was longer (9.3 vs. 6.06 months) (20). However, direct comparison is not appropriate given differences in study design, patient population, and the absence of a concurrent control group.

Exploratory subgroup analyses stratified by patient age, pre-ITI peak inhibitor titer, and timing of immunosuppressive therapy initiation revealed no clear differences in ITI success rate or time to success in this small cohort; these exploratory findings do not exclude clinically meaningful differences and should be interpreted accordingly. In this cohort, rituximab was incorporated according to two distinct approaches: concurrently at ITI initiation or after inadequate response to LD-ITI alone. This variability in rituximab timing extended across all subgroups — including those stratified by age and pre-ITI peak inhibitor titer — such that no uniform intervention was applied. The Kaplan-Meier curves therefore reflect the observed distribution of time to inhibitor eradication under heterogeneous treatment conditions rather than the effect of a single fixed protocol, and these findings should not be interpreted as demonstrating treatment efficacy or comparability with other ITI protocols.

Due to t_1/2_ testing in only six patients, although the ITI success rate was 68.8%, the tolerance rate was 37.5%. This discrepancy reflects practical challenges common to Chinese ITI studies: repeated blood sampling imposes financial burden, and geographic distance complicates sample transportation. However, during the subsequent FVIII prophylaxis phase, all 11 successfully treated patients remained free of inhibitor recurrence during a median follow-up of 24.0 months, suggesting that most patients may have achieved immunological tolerance, though this cannot be confirmed without t_1/2_ testing in all patients.

The monthly bleeding rate in our cohort (median: 0.66) was comparable to I-ITI studies (median: 0.623) (21) but higher than that reported by Li et al. (median: 0.32) (11). This discrepancy likely reflects differences in baseline patient characteristics rather than the therapeutic regimen itself. All patients in our study received on-demand factor replacement prior to ITI initiation, which resulted in more frequent bleeding episodes and progressive joint deterioration, thereby increasing the hemorrhagic burden. We propose that differences in hemostatic treatment costs between regimens would not be significant if baseline bleeding risk were equalized.

The literature reports recurrence rates of 0%–6.3% for LD-ITI + RTX-IS, and 0% for high-dose ITI. No recurrence was observed in our cohort at 24 months. Whether rituximab dose reduction affects long-term recurrence risk cannot be determined from this study given the absence of a control group and limited follow-up; prospective studies are needed. While four infection events occurred (all mild), this exceeded typical LD-ITI + RTX-IS rates. Critically, all published LD-ITI + RTX-IS studies employed IVIG prophylaxis, which our protocol lacked. Incorporating IVIG into LD-ITI + SD-RTX-IS would likely substantially reduce infection-related complications.

This study has several important limitations. First, the small single-center cohort limits statistical power and generalizability. Second, the absence of FVIII t_1/2_ testing for most patients undermines accurate assessment of true tolerance. Third, the retrospective design and absence of a control group limit causal inference. Prospective multi-center studies comparing rituximab dosing strategies are recommended.

This study reports preliminary descriptive data suggesting that LD-ITI + SD-RTX-IS may be associated with inhibitor eradication rates consistent with those described for LD-ITI + RTX-IS in prior studies, with no recurrence observed during 24-month follow-up and predominantly mild adverse events. These findings provide preliminary evidence that LD-ITI + SD-RTX-IS may represent a cost-effective approach for managing inhibitors in poor-risk patients in resource-limited settings. However, prospective controlled studies are needed before this regimen can be recommended as a new therapeutic option.

## Data Availability

The original contributions presented in the study are included in the article/**Supplementary Material**. Further inquiries can be directed to the corresponding authors.
