# Supplementary material for: Outcomes of low-dose immune tolerance induction with single-dose rituximab in severe hemophilia A: a single-center retrospective experience
Source: Front Immunol. 2026 Apr 30;17:1829007. doi: 10.3389/fimmu.2026.1829007 (PMC13171382; doi:10.3389/fimmu.2026.1829007)
Supplement: Supplementary file 1 [file Table1.docx]

Table 1. Summary of patient characteristics and outcomes.

| Patient | Age at start of ITI, y | *F8* variant | | | Interval time, mo | | Inhibitor titer, BU/mL | | | Successful Outcome (yes/no) | Time to success, mo | Follow-up period after ITI, mo | Relapse after success | Treated bleeds per mo/ joint bleeds per mo | | Adverse reactions |
| --- | --- | --- | --- | --- | --- | --- | --- | --- | --- | --- | --- | --- | --- | --- | --- | --- |
|  |  | Nucleotide change | Amino acid  change | Variant type^d^ | From inhibitor diagnosis to ITI start | From ITI start to IS use | Peak historical titer | Pre-ITI titer | Peak titer during ITI |  |  |  |  | Pre-ITI | During ITI |  |
| 01 | 41 | c.680G>A | p.(Trp227*) | Nonsense | 112.6 | 5.6 | 14.4 | 2.4 | 33.9 | No^a^ | - | 5.0 | - | 3/3 | 0.37/0.27 | Fever |
| 02 | 14 | c.709C>T | p.(Gln237*) | Nonsense | 24.9 | - | 257.0 | 70.4 | 4505.6 | No | - | 10.7 | - | 3/3 | 0.58/0.44 | - |
| 03 | 57 | c.602-139_670+1170del | p.(Gly201_Glu223del) | Large deletion | 1.4 | 3.2 | 23.7 | 23.7 | 21.1 | Yes | 9.1 | 26.9 | No | 5/4 | 0.54/0.33 | Rash |
| 04 | 40 | c.5374-?_5586+?del | - | Large deletion | 1.1 | - | 409.6 | 409.6 | 14.4 | No^a^ | - | 10.9 | - | 10/8 | 0.36/0.36 | - |
| 05 | 14 | c.144-?_265+?del | - | Large deletion | 37.1 | 4.5 | 89.2 | 7.8 | 197.1 | Yes | 14.5 | 20.8 | No | 6/6 | 0.83/0.83 | Fever |
| 06 | 4 | c.681G>A | p.(Trp227*) | Nonsense | 10.6 | - | 245.8 | 63.4 | 33.0 | Yes | 18.6 | 11.3 | No | 3/3 | 1.02/0.81 | - |
| 07 | 5 | c.389-?_601+?del | - | Large deletion | 57.1 | - | 256.0 | 24.6 | 4.7 | Yes | 10.4 | 21.8 | No | 4/4 | 0.48/0.48 | Fever |
| 08 | 15 | c.1738C>T | p.(Gln580*) | Nonsense | 6.9 | - | 108.1 | 4.5 | 9.5 | Yes | 2.8 | 28.4 | No | 2/2 | 1.07/1.07 | - |
| 09 | 13 | c.1738C>T | p.(Gln580*) | Nonsense | 6.9 | - | 119.8 | 9.8 | 11.2 | Yes | 2.8 | 28.4 | No | 4/3 | 1.43/1.43 | - |
| 10 | 4 | c.671-60_787+3601del | p.(Gly224_Pro262del) | Large deletion | 13.9 | 4.2 | 10.4 | 3.6 | 73.6 | Yes | 17.8 | 18.5 | No | 4/3 | 0.67/0.39 | - |
| 11 | 55 | c.579_582dup | p.(Leu195Serfs*6) | Frameshift | 30.1 | 11.3 | 38.2 | 1.0 | 56.3 | Yes | 15.2 | 28.9 | No | 2/1 | 0.43/0.43 | - |
| 12 | 23 | c.1538-?_1752+?del | - | Large deletion | 14.2 | - | 40.2 | 40.2 | 1.1 | Yes | 3.5 | 24.0 | No | 7/7 | 0.67/0.29 | - |
| 13 | 31 | c.1010-?_1271+?del | - | Large deletion | 87.8 | - | 200.0 | 8.5 | 952.3 | No | - | 7.9 | - | 4/1 | 0.95/0.67 | - |
| 14 | 33 | - | - | Intron 22 inversion | 0.2 | - | 137.0 | 120.3 | 256.0 | Yes | 9.3 | 22.1 | No | 3/3 | 0.86/0.43 | Nausea |
| 015 | 14 | - | - | Intron 22 inversion | 0 | 5.9 | 38.4 | 38.4 | 1024.0 | No | - | 19.0 | - | 4/4 | 0.59/0.59 | Pneumonia |
| 16 | 19 | c.1-31498_143+54del | - | Large deletion | 0.3 | - | 171.9 | 171.9 | 4.6 | Yes | 1.8 | 29.6 | No | 5/3 | 0.54/0.54 | - |
| Median  (range) | 17 (4-57) | - | - | - | 12.3 (0-112.6) | 5.1 (3.2-11.3) | 113.9 (10.4-409.6) | 24.2 (1.0-409.6) | 33.5 (1.1-4505.6) | - | 9.3 (1.8-18.6)^b^ | 22.3 (5.0-29.6)^c^ | - | 4 (2-10)/3 (1-8) | 0.66 (0.36-1.43)/0.46 (0.27-1.43) | - |

IS: Rituximab, 375 mg/m2 intravenously (maximum 600 mg/dose) × 1 dose. Prednisone dosage was 1 mg/kg (maximum 60 mg) daily for 1 month, then tapered over 3 months; Success: achieving negative inhibitor-titer (<0.6BU/mL) twice consecutively at least two weeks apart and FVIII recovery ≥66 % of expected; Non-success: failure to achieve the success criteria. Relapse: recurrence of inhibitor to ≥0.6 BU/mL after the patient had achieved success; BU/mL, Bethesda Unit/mL; y, years; mo, months; ITI, immune tolerance induction; −, not available.

1. The patient had an inhibitor titer < 5 BU/mL, with a positive response to FVIII treatment.
2. For the 11 successful patients and the 2 patients with inhibitor titers < 5 BU/mL, the median treatment duration was 10.4 (1.8-29.5) months.
3. For the 11 successful patients, the median follow-up time after ITI success was 24.0 (11.3-29.6) months.
4. All patients carried confirmed pathogenic *F8* variants.
